# Modulation of Alveolar Macrophages by Postimmunobiotics: Impact on TLR3-Mediated Antiviral Respiratory Immunity

**DOI:** 10.3390/cells11192986

**Published:** 2022-09-25

**Authors:** Mikado Tomokiyo, Fernanda Raya Tonetti, Hikari Yamamuro, Ryoko Shibata, Kohtaro Fukuyama, Nadia Gobbato, Leonardo Albarracin, Muhammad Shahid Riaz Rajoka, A. K. M. Humayun Kober, Wakako Ikeda-Ohtsubo, Julio Villena, Haruki Kitazawa

**Affiliations:** 1 Food and Feed Immunology Group, Laboratory of Animal Food Function, Graduate School of Agricultural Science, Tohoku University, Sendai 980-8576, Japan; 2 Livestock Immunology Unit, International Education and Research Center for Food Agricultural Immunology, Graduate School of Agricultural Science, Tohoku University, Sendai 980-8576, Japan; 3 Laboratory of Immunobiotechnology, Reference Centre for Lactobacilli CERELA-CONICET, San Miguel de Tucuman, Tucuman CP4000, Argentina; 4 Laboratory of Immunology, Microbiology Institute, Faculty of Biochemestry, Chemestry and Pharmacy, National University of Tucuman, Tucuman CP4000, Argentina; 5 Department of Dairy and Poultry Science, Chattogram Veterinary and Animal Sciences University, Chittagong 4225, Bangladesh

**Keywords:** porcine alveolar macrophages, TLR3, immunobiotics, postimmunobiotics, *Lactobacillus gasseri*, lung inflammatory damage

## Abstract

Beneficial microbes with immunomodulatory capacities (immunobiotics) and their non-viable forms (postimmunobiotics) could be effectively utilized in formulations towards the prevention of respiratory viral infections. In this study, novel immunobiotic strains with the ability to increase antiviral immunity in porcine alveolar macrophages were selected from a library of *Lactobacillus gasseri*. Postimmunobiotics derived from the most remarkable strains were also evaluated in their capacity to modulate the immune response triggered by Toll-like receptor 3 (TLR3) in alveolar macrophages and to differentially regulate TLR3-mediated antiviral respiratory immunity in infant mice. We provide evidence that porcine alveolar macrophages (3D4/31 cells) are a useful in vitro tool for the screening of new antiviral immunobiotics and postimmunobiotics by assessing their ability to modulate the expression *IFN-β*, *IFN-λ1*, *RNAseL*, *Mx2*, and *IL-6*, which can be used as prospective biomarkers. We also demonstrate that the postimmunobiotics derived from the *Lactobacillus gasseri* TMT36, TMT39 and TMT40 (HK36, HK39 or HK40) strains modulate the innate antiviral immune response of alveolar macrophages and reduce lung inflammatory damage triggered by TLR3 activation in vivo. Although our findings should be deepened and expanded, the results of the present work provide a scientific rationale for the use of nasally administered HK36, HK39 or HK40 to beneficially modulate TLR3-triggerd respiratory innate immune response.

## 1. Introduction

Acute viral pneumonia is one of the leading causes of related child death [1]. Severe respiratory infections induced by virus like influenza virus (IFV) and respiratory syncytial virus (RSV) results not only from the extensive viral replication in the lung but in addition to the unregulated host inflammatory response. Inflammatory alterations in respiratory viral infections have been associated with molecules such as the double-stranded RNA (dsRNA) intermediates produced during the viral replication, which are recognized by pattern recognition receptors (PRRs) expressed in immune and non-immune cells of the respiratory tract [2,3,4,5]. Among these PRRs, Toll-like receptor 3 (TLR3) stands out because it has been implicated in both protective immunity and inflammatory tissue damage during viral infections significantly affecting lung pathology as well as host survival. In this regard, it was reported that mice deficient in TLR3 are more resistant than wild-type mice to IFV infection [3]. The lungs of wild-type mice had signs of severe damage while the lungs from TLR3^−/−^ mice showed lower injuries, suggesting that TLR3 is involved in lesions induced by IFV. In contrast, it was recently described that IFV stimulates ciliary activity of the respiratory epithelium via TLR3 activation, promoting mucociliary clearance to hasten the elimination of the pathogen from the respiratory tract [6]. TLR3 can also sense the dsRNA produced during the replication of RSV [2,4]. It was shown that the lack of an appropriate regulation of TLR3 activation significantly contributes to the pulmonary immunopathology associated to RSV infection [2,4]. Furthermore, studies in BALB/c mice demonstrated that the TLR3 synthetic agonist poly(I:C), when nasally administered, induces pulmonary inflammation, bronchiolar epithelial hypertrophy, interstitial edema and altered lung function similar to the produced by RSV infection [5,7]. Coronaviruses are also able to produce dsRNA molecules during their replication and mRNA transcription [8] and it was found that the severe acute respiratory syndrome coronavirus 2 (SARS-CoV-2) is detected by the antiviral systems that sense dsRNA in the respiratory tract [9]. Of note, it was reported that the increase of cellular senescence in the respiratory tract through TLR3 contribute to SARS-CoV-2 morbidity [10]. These studies indicate that an appropriate modulation of TLR3-mediated respiratory immunity could be an interesting therapeutic target for improving the resistance to virus and reducing lung inflammatory damage.

Several studies have reported that immunomodulatory probiotic microorganisms (immunobiotics) can exert protective effects against pathogens by stimulating the mucosal immune system [11,12]. In this regard, it was recently reported that the intranasal administration of immunobiotics can improve respiratory immune responses, increasing the protection against viruses (reviewed in [13]). Studies in mice models demonstrated that nasally administered immunobiotic strains such as *Lacticaseibacillus casei* Shirota [14], *Lacticaseibacillus rhamnosus* GG [15], *Lactobacillus pentosus* S-PT84 [16], as well as *L. rhamnosus* CRL1505 and *Lactiplantibacillus plantarum* CRL1506 [17,18,19] are able to modulate cytokine production and immune cells recruitment and activation in the respiratory tract enhancing the resistance to viruses like IFV and RSV. By in vivo studies in mice, we have found that the nasal administration of *L. rhamunosus* CRL1505 increases the resistance to RSV and IFV infection via the modulation of TLR3-mediated respiratory immunity and that alveolar macrophages play an important role in this beneficial effect [17,20,21]. These studies suggest that immunobiotics or their non-viable forms (postimmunobiotics) could be effectively utilized in formulations towards the prevention of infections caused by viral respiratory infectious agents. Non-viable immunobiotics represent an interesting alternative to modulate the respiratory immunity in immunocompromised host since the use of live microorganism may represent a potential threat. In addition, the use of postimmunobiotics has practical advantages since the maintenance of viable immunobiotics requires strict temperature control, which resulted in problems such as increased production costs and resultant large delays in the utilization [22]. Interestingly, the protective effects of some postimmunobiotics were reported to be comparable to live bacteria [17,20,21]. However, the utilization of postimmunobiotics has been significantly delayed, as the detailed mechanisms leading to the protection against infections are poorly defined.

In this study, novel immunobiotic strains with the ability to increase TLR3-mediated antiviral immunity in alveolar macrophages were selected from a library of lactic acid bacteria (*Lactobacillus gasseri* strains). Postimmunobiotics derived from the most remarkable strains were also evaluated in their capacity to modulate the immune response triggered by TLR3 in alveolar macrophages and to reduce lung inflammatory damage triggered by TLR3 activation in vivo.

## 2. Materials and Methods

### 2.1. Alveolar Macrophages

Porcine alveolar macrophages (3D4/31 cells) were used in this study. Alveolar macrophages were bought from the American Type Culture Collection (CRL-2844™). The 3D4/31 cells were cultured in RPMI-1640 medium supplemented with 10% FBS (Cytiva, Marlborough, MA, USA), 100 U/mL streptomycin (Thermo Fisher Scientific, Waltham, MA, USA), and MEM-non-essential amino acid solutions (Wako, Osaka, Japan).

### 2.2. Antiviral Immune Response in Alveolar Macrophages

Porcine alveolar macrophages (1.0 × 10^5^ cells/well) were seeded into each well of 24 well plates (Corning, Corning, NY, USA) and incubated at 37 °C in the presence of 5% CO_2_ for 3 days. Then, alveolar macrophages were stimulated with freshly prepared RPMI medium supplemented with 100 ng/mL of the TLR3 agonist poly(I:C) and cultured for 0, 3, 6, 12 and 24 h. The expression of IFNs (*IFN-β* and *IFN-λ1*) and anti-viral factors (*RNAseL*, *Mx1*, *Mx2*, *PKR*, *MDA-5* and *RIG-I*) were determined by qPCR as described below.

### 2.3. Cloning of Porcine Ifns and Anti-Viral Factors

The porcine IFNs and anti-viral genes were searched from NCBI data base and designed using GENETYX SV/RC ver 13 (GENETYX, Tokyo, Japan), considering appropriate Tm-values and GC% for forward and reverse primer (Supplementary Table S1). The synthesized primers were used to amplify the gene of interest in a PCR-based Ex Taq (Takara Bio Inc., Shiga, Japan). Amplified DNA fragments were purified by using NucleoSpin Gel and PCR Clean-up (Takara Bio) and then inserted into pGEM T-Easy Vector (Promega, Madison, WI, USA) to obtain plasmids. Plasmids were mixed with 10 × KCM (K, Ca, Mg), 30% PEG (polyethylene glycol) and sterile water. The solution was used to treat *E. coli* JM109 competent cells (Takara Bio) on ice for 30 min. *E. coli* cells were incubated on TSB agar medium supplemented with 100 μg/mL (γ) Amp, X-gal (Takara Bio), and IPTG (Takara Bio) at 37 °C for 18 h. White colonies were selected and the presence or absence of insertions of the genes of interest into the vectors were confirmed by colony-PCR. Positive colonies were cultured in TSB medium supplemented with /100γ Amp at 37 °C for 18 h, and then the plasmids were extracted using Fast-Gene Plasmid Mini Kit (Nippon Genetics, Tokyo, Japan).

### 2.4. Quantitative Real-Time PCR

The total RNA of alveolar macrophages samples was isolated by using the TRIzol reagent (Invitrogen, Carlsbad, CA, USA) according to the manufacturer’s instructions. The concentration and purity of isolated RNA was determined with NanoDrop^®^ ND-1000 Spectrophotometer. The obtained RNA was converted into cDNA by using the PrimeScript RT reagent Kit (Takara Bio) by following the manufactures instructions. The quantitative real time PCR was performed in a CFX Connect Real-time PCR System (Bio-rad, Hercules, CA, USA) using the platinum SYBR Green (Invitrogen) according to the manufacturer’s recommendations. The primers used were listed in Supplementary Table S1. The thermal cycling conditions were 50 °C for 2 min; 95 °C for 5 min; 95 °C for 15 s; 60 °C for 30 s and 72 °C for 30 s for 39 cycles. The β-actin, which is stably expressed in various tissues of pigs, was used as a housekeeping gene. The expression level of mRNA was calculated using the calibration curve obtained from serially diluted plasmids, which was normalized by the expression level of β-actin in each sample, and then expressed as relative with the control set as 1.

### 2.5. Microorganisms

Thirty-six *Lactobacillus gasseri* strains isolated from the human intestinal tract were kindly provided by Ishibashi Hardware CO., Ltd. (Fukuoka, Japan) (Supplementary Table S2). For experiments, *L. gasseri* strains were cultured in MRS medium (Becton Dickinson, Franklin Lakes, NJ, USA) at 37 °C for 16 h and washed three times with PBS. For the obtention of non-viable lactobacilli heat-treatment was used as previously described [20,21]. Briefly, lactobacilli were heat-treated on a heat block at 65, 90 or 121 °C for 30 min. The viable and non-viable bacteria solutions were suspended in PBS at a concentration of 2.5 × 10^9^ cells/mL and kept at –20 °C for further experiments.

### 2.6. Modulation of Alveolar Macrophages’ Immune Response by Lactobacilli

Porcine alveolar macrophages (1.0 × 10^5^ cells/well) were seeded into each well of 24 well plates and incubated overnight at 37 °C in the presence of 5% CO_2_. Viable or heat-killed lactic acid bacterial strains (6.6 × 10^7^ cells/mL) were added and cultured at 37 °C for 2 days. After incubation, alveolar macrophages were washed three times with PBS, stimulated with RPMI medium supplemented with 100 ng/mL of poly (I:C) and cultured for 3 to 12 h. The expression analysis of various immune factors including IFNs, anti-viral factors and negative regulators of the TLR signaling pathway was performed by qPCR as described above.

### 2.7. Phagocytosis

Porcine alveolar macrophages (3.0 × 10^5^ cells/well) were seeded in to 24 well plates and cultured overnight at 37 °C in the presence of 5% CO_2_. After change to fresh medium, 5(6)-Carboxyfluorescein diacetate (CFDA)-labeled heat-killed lactic acid bacterial strains (2.0 × 10^8^ cells/mL) were added and cultured at 37 °C for 30 min. After incubation, the cells were washed three times with PBS and harvested from the plates using tryptic solutions (0.25% trypsin, 0.02% EDTA in PBS). Cell pellets were fixed with paraformaldehyde solution (1% paraformaldehyde in PBS) at room temperature for 15 min in dark. Cell suspensions were analyzed using FACS Accuri C6 (Becton Dickinson). Data obtained were analyzed using FlowJo software. Because differences in fluorescence intensity were observed among strains, the data were corrected for mean fluorescence intensity.

For light microscopy analysis, alveolar macrophages (1.0 × 10^6^ cells/well) were seeded in wells of glass-covered 6 well plates and cultured overnight at 37 °C in the presence of 5% CO_2_. Cells were stimulated with heat-killed lactobacilli (1.3 × 10^8^ cells/mL) for 30 min. After fixation for 30 min with methanol and staining with Giemsa stain (Wako), phagocytosis was observed by light microscopy.

### 2.8. Westen Blotting Analysis

Porcine alveolar macrophages were stimulated with heat-killed lactic acid bacterial strains as described above. Then, the cells were washed three times with PBS and subsequently stimulated with RPMI medium supplemented with 100 ng/mL of poly (I:C). At 0, 30, 60, 90 and 120 min post-stimulation, the cells were washed with PBS and lysed by adding 200 μL of RIPA Lysis buffer containing protease and inhibitors of phosphates (ATTO, Tokyo, Japan). The lysed cells were transferred to Eppendorf tubes (1.5 mL) and centrifugated at 15,000 rpm for 5 min at 4 °C. Protein concentrations in supernants were determined with the BCA assay kit (Pierce, Rockford, IL, USA). Samples containing proteins (8 μg) were loaded on 10% SDS-polyacrylamide gels and then transferred to PVDF membranes (Trans-Blot Turbo RTA Transfer PVDF Kit, Bio-Rad). The membranes were incubated with Everyblot blocking buffer (Bio-Rad) for 5 min and then treated with primary and secondary antibodies. Interferon regulatory factor 3 (IRF-3) and TNF receptor-associated factor (TRAF3) were studied with Phospho-IRF3 (Ser396) (Cat. #4947) rabbit monoclonal antibody from Cell Signaling Technology (Beverly, MA, USA) and TRAF3 rabbit polyclonal antibody (Cat. #4729), respectively. A 1,000 times dilution of antibodies were used at 4 °C. After washing with TBS-T buffer and incubated with anti-rabbit IgG, AP-linked antibody (Cat. #7054) for 1h at room temperature, the membranes were spread with 200 ul of ECF substrate (GE Healthcare Japan, Tokyo, Japan) to detect optical protein bands and photographed by ChemiDoc TouchMP (Bio-Rad). Proteins bands were estimated from the peak area of densitogram by Image Lab software (Bio-Rad).

### 2.9. Experimental Aniamls and Treatments

Infant (3-week-old) BALB/c mice were obtained from the closed colony kept at CERELA (San Miguel de Tucumán, Argentina). Mice were housed in plastic cages at room temperature. Five to six animals were used per group for each time point for the assays performed. Non-viable *L. gasseri* TMT36 (HK36), TMT39 (HK39), or TMT40 (HK40) strains were administered through the nasal route to infant mice for two consecutive days (10^8^ cells/mouse/day) in 50 μL of PBS [20,21]. The treated groups and the PBS-treated control group were fed a conventional balanced diet *ad libitum*.

This study was carried out in strict accordance with the recommendations in the Guide for the Care and Use of Laboratory Animals of the Guidelines for Animal Experimentation of CERELA. The CERELA Institutional Animal Care and Use Committee prospectively approved this research under the protocol BIOT-CRL-18. All efforts were made to minimize the number of animals and their suffering. No signs of discomfort or pain were observed before mice reached the endpoints. No deaths were observed before mice reached the endpoints.

### 2.10. In Vivo Poly(I:C) Administration

Poly(I:C), the TLR3 agonist, was administered as described previously [20,21]. Briefly, two days after the last day of treatments with HK36, HK39, or HK40, mice received 100 μL of PBS containing 250 μg poly(I:C) (equivalent to 10 mg/kg body weight) through the nasal route. Control mice received 100 μL of PBS. Animals received three doses of poly(I:C) or PBS with 24 h rest period between each administration.

Broncho-alveolar lavages (BAL) samples were obtained as described previously [17,23]. Albumin content was determined colorimetrically using an albumin diagnostic kit (Wiener Lab, Buenos Aires, Argentina). BAL lactate dehydrogenase (LDH) activity was assessed with the Wiener reagents and procedures (Wiener Lab).

IFN-β (Mouse IFN-beta ELISA Kit), IFN-γ (Mouse IFN-gamma Quantikine ELISA Kit), IL-6 (Mouse IL-6 Quantikine ELISA Kit), IL-10 (Mouse IL-10 Quantikine ELISA Kit), IL-12 (Mouse IL-12 p70 DuoSet ELISA), tumor necrosis factor (TNF)-α (Mouse TNF-α 236 ELISA Kit) and IL-27 (Mouse IL-27 p28/IL-30 Quantikine ELISA Kit) concentrations in BAL samples were measured with commercially ELISA technique kits following the manufacturer’s recommendations (R&D Systems, MN, USA). CCL2 (Mouse MCP1 ELISA Kit (ab208979), and chemokine KC (or CXCL1) were measured with commercially available ELISA technique kits following the manufacturer’s recommendations (Abcam).

### 2.11. Alveolar Macrophages Primary Cultures

Primary cultures of murine alveolar macrophages were performed as described previously [21]. Briefly, alveolar macrophages obtained from infant mice via BAL samples were transferred to new sterile tubes, washed twice in sterile PBS, and resuspended in RPMI 1640 medium with 10% FBS, 1 mM L-glutamine, and 100 U/mL penicillin-streptomycin. BAL cells were seeded in 24-well plates at a density of 10^5^ cells/well and incubated for 2 h at 37 °C in 5% CO_2_ to promote adherence. Non-adherent cells were washed and maintained in culture in RPMI 1640 medium with 10% FBS, 1 mM L-glutamine, and 100 U/mL penicillin-streptomycin at 37 °C in 5% CO_2_ for 24 h before stimulation. Alveolar macrophages obtained from control and HK36-, HK39-, or HK40-treated mice were collected, and the primary cultures were stimulated with poly(I:C) (50 ug/mL). Supernatants were collected twenty-four hours after TLR3 activation for cytokines analysis commercially available ELISA technique kits as described above.

### 2.12. Statistical Analysis

Experiments were performed in triplicate and results were expressed as mean ± standard deviation (SD). After verification of the normal distribution of data, 2-way ANOVA was used. Tukey’s test (for pairwise comparisons of the means) was used to test for differences between the groups. Differences were considered significant at *p* < 0.05.

## 3. Results

### 3.1. Response of Porcine Alveolar Macrophages to Poly(I:C) Challenge

**Figure 1:**
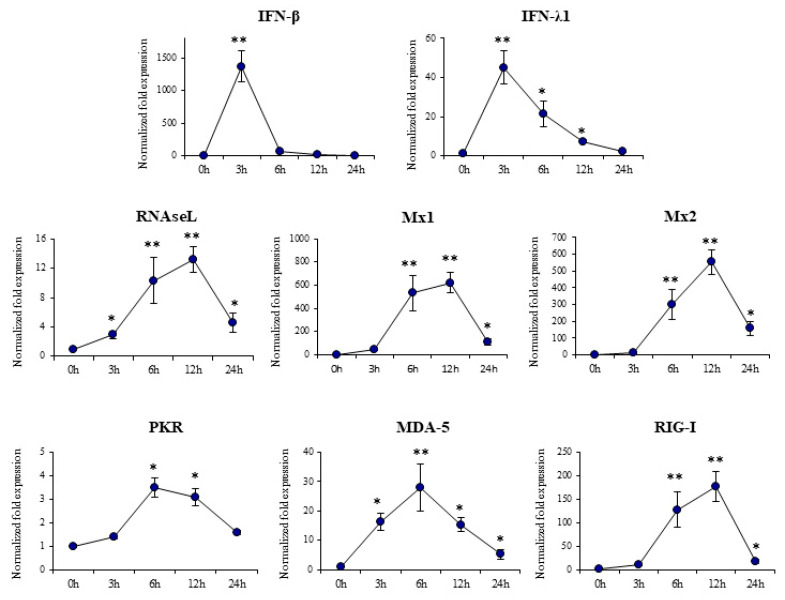
Expression of interferons and interferon-induced antiviral genes in porcine alveolar macrophages after the challenge with the Toll-like receptor 3 (TLR3) agonist poly(I:C). The expression of *IFN-β*, *IFN-λ1*, *RNAseL*, *Mx1*, *Mx2*, *PKR*, *MDA-5* and *RIG-I* were analyzed by qPCR at the indicated time points after poly(I:C) challenge. The results represent data from three independent experiments. Significant differences when compared to unchallenged control porcine alveolar macrophages (time 0 h). * (*p* < 0.05), ** (*p* < 0.01).

In order to evaluate gene expression changes induced by poly(I:C) in porcine alveolar macrophages qPCR was performed. The expression of *IFN-β*, *IFN-λ1*, and the antiviral factors *RNAseL*, *Mx1*, *Mx2*, *PKR*, *MDA-5* and *RIG-I* was evaluated at different time points after poly(I:C) challenge as shown in Figure 1.

Type I (*IFN-β*) and type III (*IFN-λ1*) IFNs were significantly increased after the activation of TLR3 and peaked at hour 3 post-stimulation. The expression of *IFN-β* in porcine alveolar macrophages returned to basal values at hour 6 while the mRNA levels of *IFN-λ1* decreased gradually until hour 24. The expressions of the interferon-stimulated genes (ISGs) *Mx1*, *Mx2* and *RNAseL* was increased in alveolar macrophages after poly(I:C) challenge, with a peak in the three of them at hour 12 (Figure 1). The expression of the anti-viral pattern recognition receptors (PRRs) *PKR*, *MDA-5* and *RIG-I* had a peak between hours 6 and 12 after the stimulation with poly(I:C) (Figure 1). These findings indicate that 3D4/31 alveolar macrophages are capable of responding to TLR3 activation by poly(I:C) by increasing expression of type I and type III IFNs and the subsequent up-regulation of antiviral ISGs.

### 3.2. Selection of New Immunobiotics with the Ability to Modulate Porcine Alveolar Macrophages

**Figure 2:**
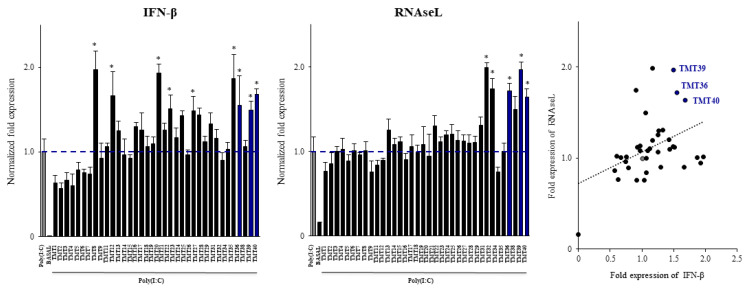
Effect of viable lactic acid bacteria strains on the expression of interferon and interferon-induced antiviral genes in porcine alveolar macrophages after the activation of Toll-like receptor 3 (TLR3). Alveolar macrophages were stimulated with viable bacteria and then challenged with the TLR3 agonist poly(I:C). The expression of *IFN-β* and *RNAseL* were analyzed by qPCR after 12 h of TLR3 activation. Non-bacteria treated alveolar macrophages challenged with poly(I:C) were used as controls. The results represent data from three independent experiments. Significant differences were observed when compared to poly(I:C)-challenged control porcine alveolar macrophages. * (*p* < 0.05). Correlation between the fold expression of *IFN-β* and *RNAseL* by a linear regression function was performed.

We next aimed to select new immunobiotic strains with the capacity to modulate the response of porcine alveolar macrophages to the activation of TLR3. For this purpose, a library of 36 lactic acid bacterium strains were evaluated. Each strain was used to stimulate 3D4/31 alveolar macrophages for 2 days before the stimulation with poly(I:C). The expression of *IFN-β* and *RNAseL* were used to select the strains with immunomodulatory potential (Figure 2). The results showed that among the 36 strains, only 9 were able to significantly increase the expression of *IFN-β* while only 5 up-regulated *RNAseL* expression. Correlation between the fold expression of *IFN-β* and *RNAseL* by a linear regression function demonstrated that the strains with the most remarkable effect on porcine alveolar macrophages were *L. gasseri* TMT36, TMT39 and TMT40 (Figure 2).

### 3.3. Selection of Postimmunobiotics with the Ability to Modulate Porcine Alveolar Macrophages

**Figure 3:**
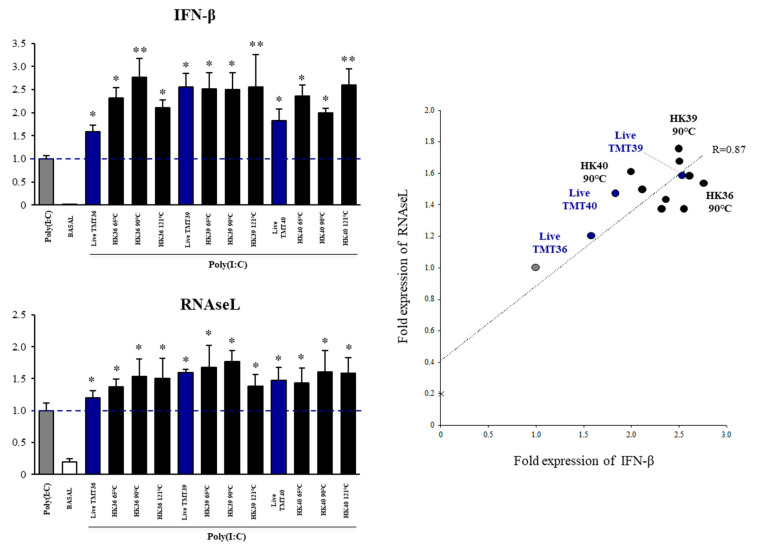
Effect of non-viable lactic acid bacteria strains on the expression of interferon and interferon-induced antiviral genes in porcine alveolar macrophages after the activation of Toll-like receptor 3 (TLR3). Alveolar macrophages were stimulated with viable or non-viable bacteria and then challenged with the TLR3 agonist poly(I:C). The expression of *IFN-β* and *RNAseL* were analyzed by qPCR after 12 h of TLR3 activation. Non-bacteria treated alveolar macrophages challenged with poly(I:C) were used as controls. The results represent data from three independent experiments. Significant differences were observed to poly(I:C)-challenged control porcine alveolar macrophages. * (*p* < 0.05); ** (*p* < 0.01). Correlation between the fold expression of *IFN-β* and *RNAseL* by a linear regression function was performed.

Our previous studies in mice models demonstrated that non-viable bacteria derived from immunobiotics strain may retain the immunomodulatory activities [17,20,21]. Thus, we next aimed to evaluate whether *L. gasseri* TMT36, TMT39 and TMT40 retained their ability to differentially modulate the response of porcine alveolar macrophages after their heat treatment. Each strain was heat-treated at 65 °C, 90 °C or 121 °C for 30 min and their capacities to modulate the expression of *IFN-β* and *RNAseL* in alveolar macrophages challenged with poly(I:C) were compared with viable strains (Figure 3). Of note, the expression of *IFN-β* was significantly increased in heat-killed *L. gasseri* TMT36 (HK36) when compared to the viable strain, with the 90 °C treatment. Similarly, *IFN-β* expression was improved in alveolar macrophages stimulated with heat-killed *L. gasseri* TMT40 (HK40) when compared to the viable strain, with the 121 °C treatment (Figure 3). All other HK treatments were as efficient as their respective viable strains in increasing the type I IFNs expression in poly(I:C)-stimulated porcine alveolar macrophages. In addition, all the HK treatments preserved the capacity of viable *L. gasseri* strains to increase the expression of *RNAseL* (Figure 3). A correlation analysis between the fold expression of *IFN-β* and *RNAseL* by a linear regression function was performed to find out the most remarkable HK treatments (Figure 3). Notably, we found that strains subjected to 90 °C for 30 min were the ones with the optimal capacity to enhance the antiviral factors. Thus, HK36, HK39 and HK40 obtained by 90 °C treatment were selected as potential postimmunobiotics for further studies.

### 3.4. Effect of Postimmunobiotics in Alveolar Macrophages Activities

**Figure 4:**
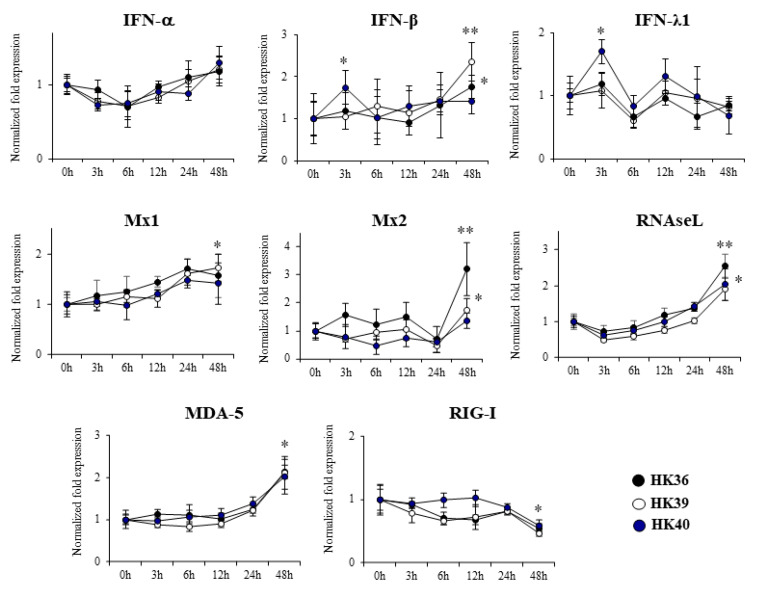
Effect of non-viable *Lactobacillus gasseri* strains on the expression of interferons and interferon-induced antiviral genes in porcine alveolar macrophages. Alveolar macrophages were stimulated with non-viable *L. gasseri* TMT36 (HK36), TMT39 (HK39), or TMT40 (HK40) and the expression of *IFN-α*, *IFN-β*, *IFN-λ1*, *RNAseL*, *Mx1*, *Mx2*, *MDA-5* and *RIG-I* were analyzed by qPCR at the indicated time points. The results represent data from three independent experiments. Significant differences were observed to unchallenged control porcine alveolar macrophages (time 0 h). * (*p* < 0.05), ** (*p* < 0.01).

**Figure 5:**
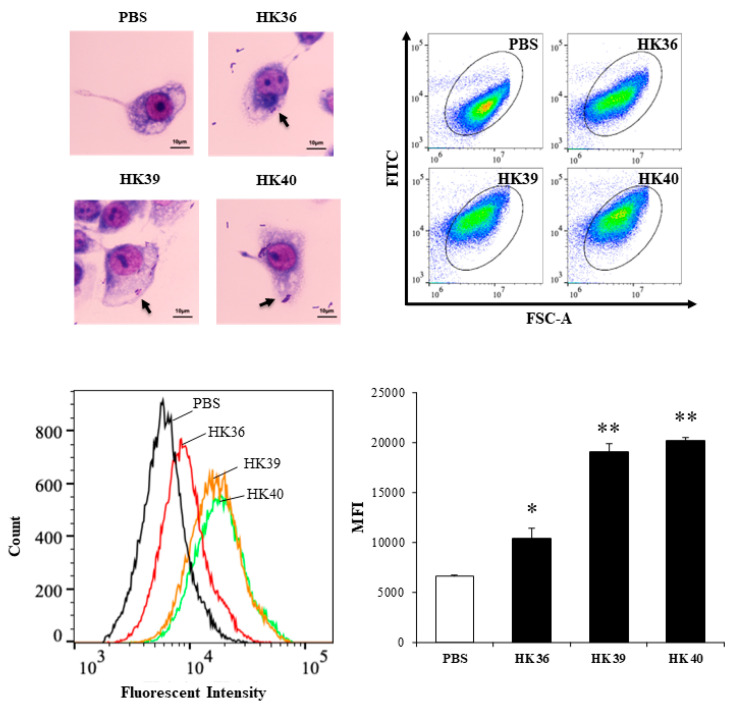
Phagocytosis of non-viable *Lactobacillus gasseri* strains by porcine alveolar macrophages. Alveolar macrophages were stimulated with non-viable *L. gasseri* TMT36 (HK36), TMT39 (HK39), or TMT40 (HK40) and phagocytosis was evaluated by flow cytometry and microscopic analysis. The results represent data from three independent experiments. Significant differences were observed when compared to control porcine alveolar macrophages treated with PBS. * (*p* < 0.05), ** (*p* < 0.01).

We next aimed to investigate the effect of HK36, HK39 and HK40 on the expression of immune factors (Figure 4) and phagocytic activity (Figure 5) of porcine alveolar macrophages. No significant changes were observed in the expression of *IFN-α* when alveolar macrophages treated with HK36, HK39 or HK40 were analyzed after 3-, 6-, 12-, 24- or 48-h post-stimulation (Figure 4). In contrast, the HK40 treatment was able to significantly increase the expression of *IFN-β* and *IFN-λ1* at hour 3 post-stimulation while HK36 and HK39 enhanced *IFN-β* RNAm levels of at hour 48. The three treatments HK36, HK39 and HK40 were capable to up regulate the expression of *RNAseL* and *MDA-5* and to down regulate the expression of RIG-I at hour 48 post-stimulation while only HK36 and HK39 significantly increased *Mx1* and *Mx2* at hour 48 (Figure 4).

As shown in Figure 5, the three postimmunobiotics HK36, HK39 and HK40 were efficiently phagocyted by porcine alveolar macrophages. A higher number of phagocyted HK39 and HK40 was observed when compared to HK36.

### 3.5. Effect of Postimmunobiotics in the Response of Alveolar Macrophages to TLR3 Activation

**Figure 6:**
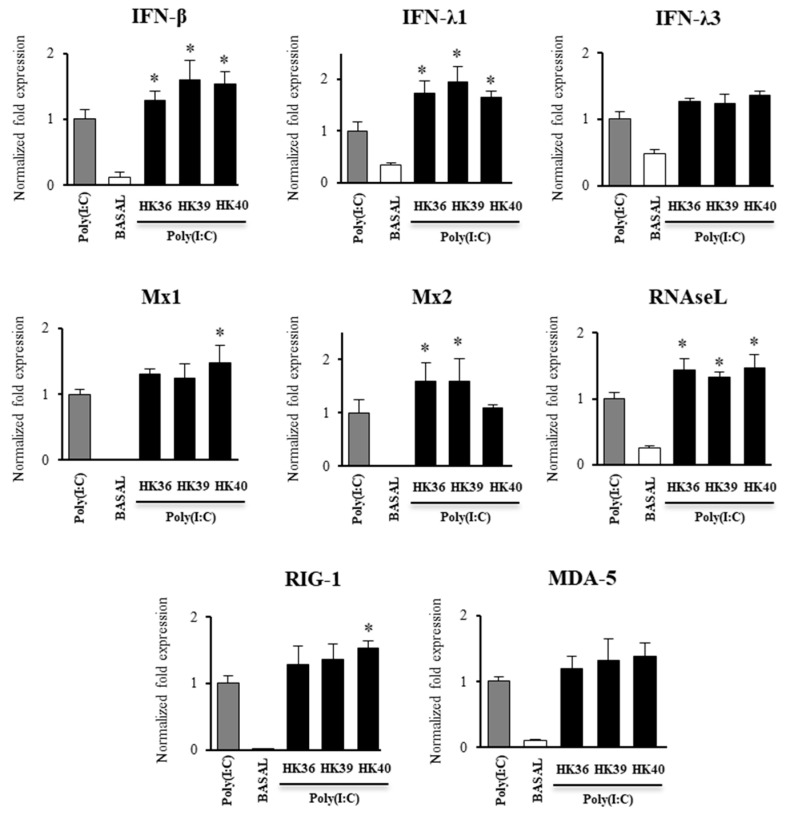
Effect of non-viable *Lactobacillus gasseri* strains on the expression of interferons and interferon-induced antiviral genes in porcine alveolar macrophages after the activation of Toll-like receptor 3 (TLR3). Alveolar macrophages were stimulated with non-viable *L. gasseri* TMT36 (HK36), TMT39 (HK39), or TMT40 (HK40) and then challenged with the TLR3 agonist poly(I:C). The expression of *IFN-β*, *IFN-λ1*, *IFN-λ3, RNAseL*, *Mx1*, *Mx2*, *MDA-5* and *RIG-I* were analyzed by qPCR after 12 hours of TLR3 activation. Non-lactobacilli treated alveolar macrophages challenged with poly(I:C) were used as controls. The results represent data from three independent experiments. Significant differences were observed when compared to polyI(:C)-challenged control porcine alveolar macrophages. * (*p* < 0.05).

The effect of HK36, HK39 and HK40 on the expression of immune factors in porcine alveolar macrophages after the stimulation whit the TLR3 agonist poly(I:C) was investigated. The three postimmunobiotic treatments were able to increase the expression of *IFN-β*, *IFN-λ1* and *RNAseL* while no significant differences were observed in the expression of *IFN-λ3* when these groups were compared to control alveolar macrophages challenged only with poly(I:C) (Figure 6).

**Figure 7:**
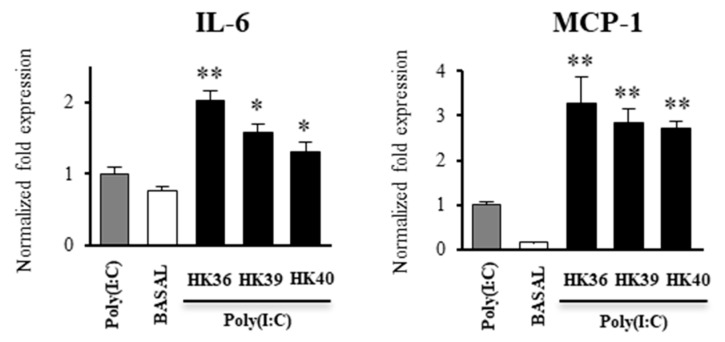
Effect of non-viable *Lactobacillus gasseri* strains on the expression of inflammatory cytokines genes in porcine alveolar macrophages after the activation of Toll-like receptor 3 (TLR3). Alveolar macrophages were stimulated with non-viable *L. gasseri* TMT36 (HK36), TMT39 (HK39), or TMT40 (HK40) and then challenged with the TLR3 agonist poly(I:C). The expression of *IL-6* and *MCP-1* were analyzed by qPCR after 12 hours of TLR3 activation. Non-lactobacilli treated alveolar macrophages challenged with poly(I:C) were used as controls. The results represent data from three independent experiments. Significant differences were observed when compared to polyI(:C)-challenged control porcine alveolar macrophages. * (*p* < 0.05), ** (*p* < 0.01).

Only the treatment with HK40 up regulated the expression of *Mx1* while HK36 and HK39 significantly enhanced the RNAm levels of *Mx2* (Figure 6). HK40 increased the expression of *RIG-I* while none of the treatments was able to induce changes in the expression of *MDA-5* (Figure 6). The expression levels of the inflammatory factors *IL-6* and *MCP-1* were also assessed in alveolar macrophages challenged with poly(I:C) (Figure 7). It was observed that the three postimmunobiotic treatments were able to significantly increase the expression of *IL-6* and *MCP-1* in porcine alveolar macrophages after the activation of TLR3.

**Figure 8:**
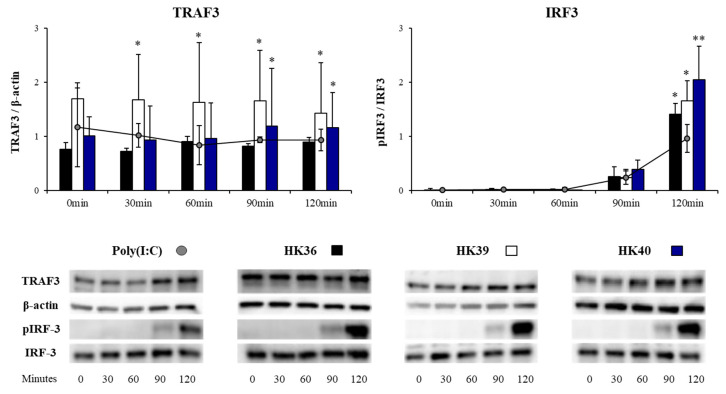
Effect of non-viable *Lactobacillus gasseri* strains on signaling pathway in porcine alveolar macrophages after the activation of Toll-like receptor 3 (TLR3). Alveolar macrophages were stimulated with non-viable *L. gasseri* TMT36 (HK36), TMT39 (HK39), or TMT40 (HK40) and then challenged with the TLR3 agonist poly(I:C). The proteins from lysed cells were extracted at the indicated time points and separated by SDS-PAGE. Western-blot was performed to quantify TRAF3/β-actin and p-IRF3/IRF3 ratios. Intensities of proteins bands were calculated from peak area of densitogram by using image Lab software. Non-lactobacilli treated alveolar macrophages challenged with poly(I:C) were used as controls. The results represent data from three independent experiments. Significant differences were observed when compared to polyI(:C)-challenged control porcine alveolar macrophages. * (*p* < 0.05), ** (*p* < 0.01).

In order to further confirm the ability of HK36, HK39 and HK40 on the TLR3 signaling in porcine alveolar macrophages, we next performed western-blot analysis to quantify TRAF3/β-actin and p-IRF3/IRF3 ratios (Figure 8).

As expected, the challenge of porcine alveolar macrophages with poly(I:C) significantly increased the levels of TRAF3 from min 30 to 120, as well as the p-IRF3/IRF3 ratio, indicating the activation of the TLR3 pathway (Figure 8). The levels of TRAF3 were significantly increased in alveolar macrophages treated with HK39 from min 30 to 120, while this factor was increased from min 90 to 120 in macrophages treated with HK40. Of note, TRAF3 was not different from control cells in alveolar macrophages stimulated with HK36. The three HK36, HK39 and HK40 treatments enhanced the p-IRF3/IRF3 ratio when compared to control cells at min 120 (Figure 8).

**Figure 9:**
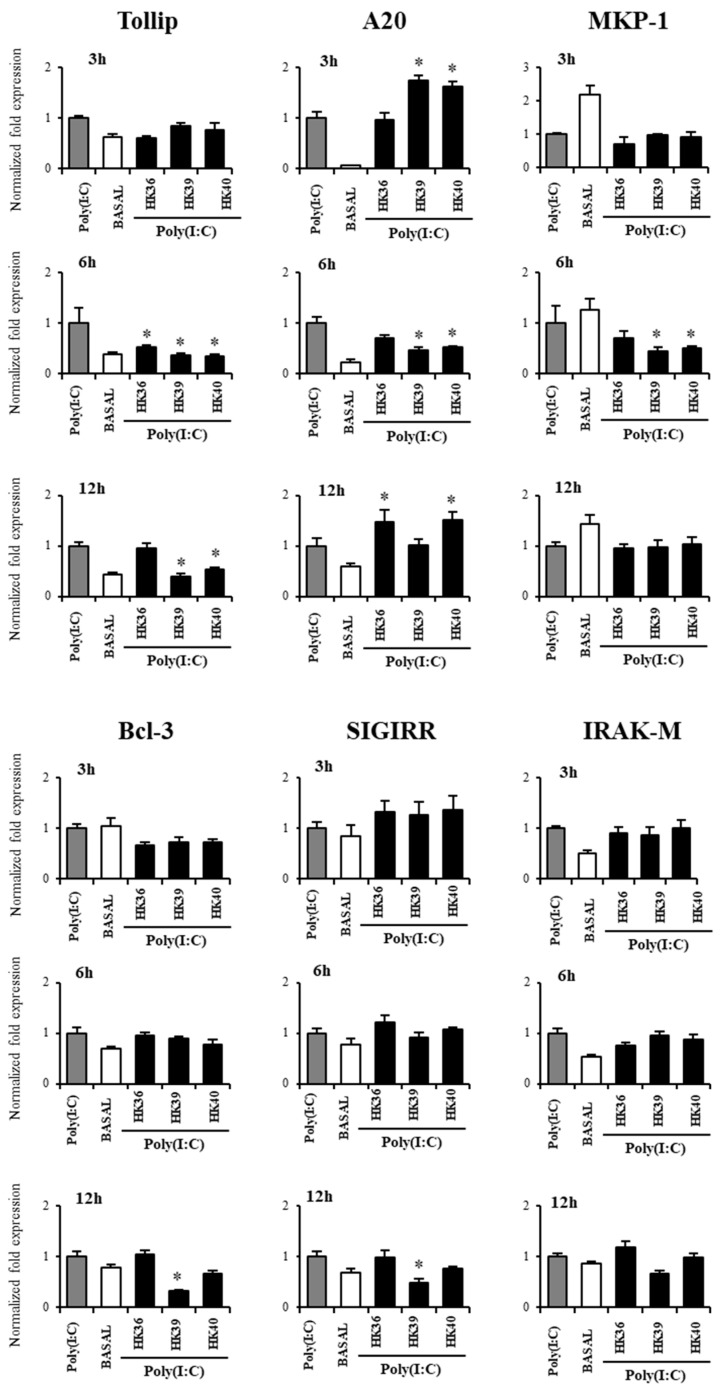
Effect of non-viable *Lactobacillus gasseri* strains on the expression of negative regulators of the Toll-like receptor (TLR) signaling pathway in porcine alveolar macrophages after the activation of TLR3. Alveolar macrophages were stimulated with non-viable *L. gasseri* TMT36 (HK36), TMT39 (HK39), or TMT40 (HK40) and then challenged with the TLR3 agonist poly(I:C). The expression of *Tollip, A20, MKP-1, Bcl-3, SIGIRR* and *IRAK-M* were analyzed by qPCR. Non-lactobacilli treated alveolar macrophages challenged with poly(I:C) were used as controls. The results represent data from three independent experiments. Significant differences were observed when compared to polyI(:C)-challenged control porcine alveolar macrophages. * (*p* < 0.05).

Our previous studies demonstrated that immunobiotics modulate the TLR3 signaling through the induction of changes in the expression of negative regulators of the TLR signaling pathway [24,25]. Thus, we next evaluated the expressions of *Tollip*, *A20*, *MKP-1*, *Bcl-3*, *SIGIRR* and *IRAK-M* in alveolar macrophages stimulated with HK36, HK39 or HK40 and then challenged with poy(I:C) (Figure 9).

No significant differences were observed in the expression of *Bcl-3*, *SIGIRR* and *IRAK-M* in alveolar macrophages treated with HK36, HK39 or HK40 when compared to control cells, except for *Bcl-3* and *SIGIRR* that were down regulated by HK39 at hour 12 post-poly(I:C) stimulation (Figure 9). HK39 and HK40 reduced the expression of *MKP-1* at hour 6 and *Tollip* at hour 12 while the three treatments down regulated *Tollip* expression at hour 6 post-poly(I:C) stimulation. HK39 and HK40 increased the expression of *A20* at hour 3 while both treatments reduced the expression of this negative regulator of the TLR signaling pathway at hour 6 (Figure 9). Of note, HK36 and HK40 up regulated *A20* expression in porcine alveolar macrophages challenged with poly(I:C) at hour 12.

### 3.6. Effect of Postimmunobiotics in the Respiratory Innate Immune Response Triggered by TLR3 Activation In Vivo

**Figure 10:**
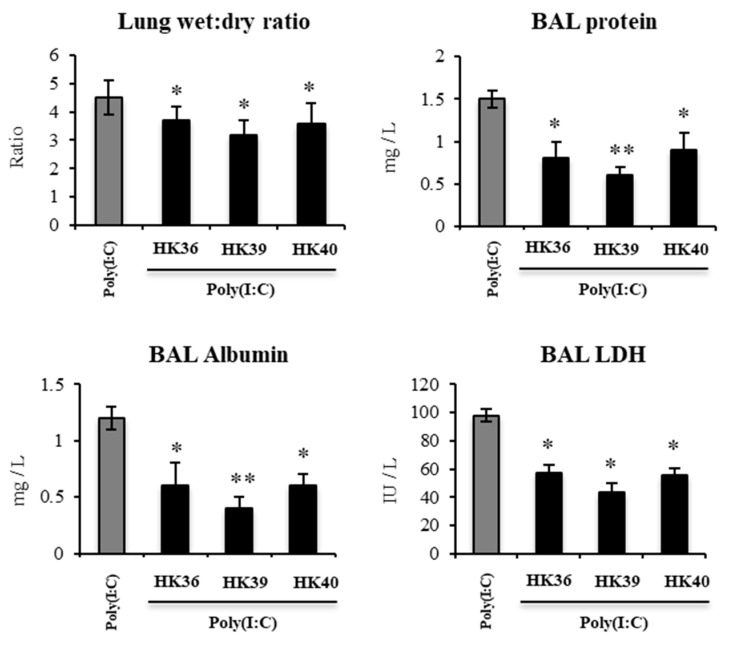
Effect of non-viable *Lactobacillus gasseri* strains on the lung tissue injury induced by the activation of Toll-like receptor 3 (TLR3). Infant mice were nasally primed with non-viable *L. gasseri* TMT36 (HK36), TMT39 (HK39), or TMT40 (HK40) during two consecutive days and challenged with three once-daily doses of poly(I:C). Non-lactobacilli treated mice challenged with poly(I:C) were used as controls. Lung wet: dry ratio, broncho-alveolar lavages (BAL) concentrations of albumin and proteins, and lactate dehydrogenase (LDH) activity were evaluated 2 days after the last poly(I:C) administration. The results represent data from three independent experiments. Significant differences were observed when compared to the control group * (*p* < 0.05), ** (*p* < 0.01).

Considering the remarkable ability of HK36, HK39 or HK40 to differentially modulate the antiviral immune response of porcine alveolar macrophages we next aimed to evaluate whether these postimmunobiotics could modulate the respiratory innate immune response triggered by TLR3 activation in vivo. For this purpose, infant mice were nasally primed with HK36, HK39 or HK40 and then challenged with poly(I:C). Two days after TLR3 activation, the ability of postimmunobiotic treatments to protect the lungs against the inflammatory damage was performed assessing lung wet:dry ratio and biochemical parameters in broncho-alveolar lavages (BAL) (Figure 10). The nasal administration of poly(I:C) to infant mice significantly increased the lung wet:dry ratio as well as the levels of proteins, albumin and LDH in BAL samples, indicating edema, alteration of the permeability of the bronchoalveolar-capillarity barrier and respiratory cellular damage. Of note, the treatment of mice with HK36, HK39 or HK40 significantly reduced lung wet:dry ratio and the levels of biochemical parameters in BAL after the challenge with poly(I:C), when compared to control mice (Figure 10).

**Figure 11:**
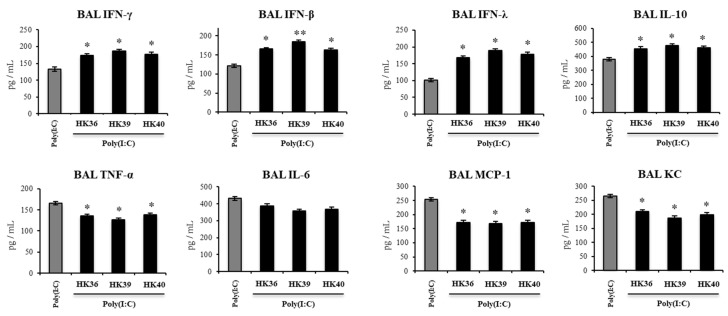
Effect of non-viable *Lactobacillus gasseri* strains on the respiratory immune response induced by the activation of Toll-like receptor 3 (TLR3). Infant mice were nasally primed with non-viable *L. gasseri* TMT36 (HK36), TMT39 (HK39), or TMT40 (HK40) during two consecutive days and challenged with three once-daily doses of poly(I:C). Non-lactobacilli treated mice challenged with poly(I:C) were used as controls. Broncho-alveolar lavages (BAL) concentrations of interferon (IFN)- γ, IFN-β, IFN-λ, interleukin (IL)-10, IL-6, tumor necrosis factor (TNF)-α, MCP-1 and KC were evaluated 2 days after the last poly(I:C) administration. The results represent data from three independent experiments. Significant differences were observed when compared to the control group * (*p* < 0.05), ** (*p* < 0.01).

The levels of IFN-β, IFN-γ, IFN-λ, TNF-α, IL-6, KC, MCP-1 and IL-10 in BAL were also determined after the administration of poly(I:C) in infant mice treated with HK36, HK39 or HK40 (Figure 11). The administration of the TLR3 agonist induced significant increases in the levels of BAL IFNs, inflammatory cytokines and chemokines and IL-10 as previously reported [21]. The three postimmunobiotic treatments increased the levels of IFN-β, IFN-γ and IFN-λ in the respiratory tract of infant mice challenged with poly(I:C). In addition, HK36, HK39 and HK40 were equally effective to reduce the levels of BAL TNF-α, KC and MCP-1 and increase BAL IL-10 when compared to controls (Figure 11). No differences were found between control mice and animals treated with HK36, HK39 or HK40 when the respiratory levels of IL-6 were analyzed after the activation of TLR3.

**Figure 12:**
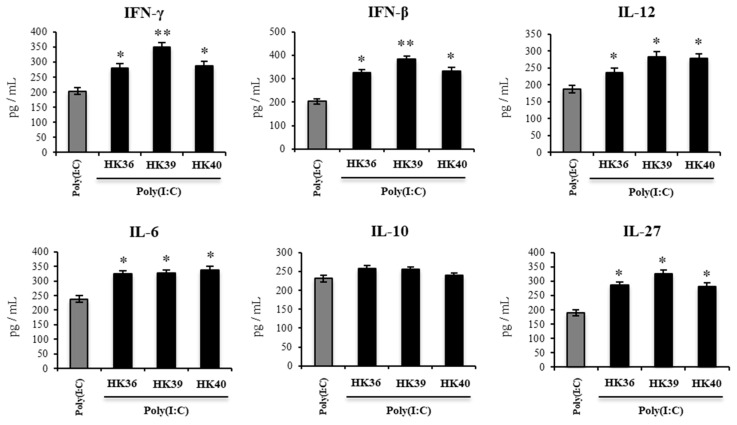
Effect of non-viable *Lactobacillus gasseri* strains on the immune response induced by the activation of Toll-like receptor 3 (TLR3) in murine alveolar macrophages. Infant mice were nasally primed with non-viable *L. gasseri* TMT36 (HK36), TMT39 (HK39), or TMT40 (HK40) during two consecutive days. Non-lactobacilli treated mice were used as controls. Broncho-alveolar lavages (BAL) were collected to perform primary cultures of alveolar macrophages of the different groups. Macrophages were challenged in vitro with poly(I:C). The levels of interferon (IFN)-β, IFN-γ, interleukin (IL)-6, IL-10, IL-12, and IL-27 were evaluated on alveolar macrophage supernatants after 24 h. The results represent data from three independent experiments. Significant differences were observed when compared to the control group * (*p* < 0.05), ** (*p* < 0.01).

Finally, considering that the in vitro results in porcine alveolar macrophages suggested that this immune cell population is relevant for the immunomodulatory effects of HK36, HK39 and HK40, the changes induced by these postimmunobiotic treatments in murine alveolar macrophages cytokine profiles in response to poly(I:C) was evaluated. For this purpose, primary cultures of alveolar macrophages from the control or postimmunobiotic-treated infant mice were prepared, and cells were challenged in vitro with poly(I:C) (Figure 12). The production of IFN-β, IFN-γ, IL-6, IL-12, IL-10 and IL-27, was evaluated in the supernatants of alveolar macrophages cultures after 24 hs of TLR3 activation. All the cytokines evaluated except for IL-10 were significantly higher in murine alveolar macrophages cultures obtained from postimmunobiotic-treated infant mice when compared to the controls (Figure 12). Of note, the HK39 treatment was the most efficient to increase the production of IFN-β and IFN-γ.

## 4. Discussion

Our previous studies in mice models have demonstrated that alveolar macrophages play an important role in the ability of nasally administered *L. rhamunosus* CRL1505 to modulate the antiviral innate immune response triggered by TLR3 activation and to increase the resistance against viral infections [17,20,21]. For this reason, we hypothesized that the in vitro study of the interaction between alveolar macrophages and lactobacilli could be a valuable tool to select strains with the potential to improve respiratory antiviral immunity. In this work, we evaluated this hypothesis by studying the interaction of porcine alveolar macrophages with strains of the species *L. gasseri*, in the context of the immune response triggered by the activation of TLR3.

On the one hand, the 3D4/31 cell line was selected to perform the studies of macrophages/lactobacilli interactions as it obviates the need for primary cultures. This cell line derived from porcine alveolar macrophages have been shown to be of value for the in vitro study of antiviral immunity [26]. Of note, pigs are more similar to humans than rodents in terms of anatomy and physiology, making pigs an attractive option for modeling human diseases [27]. In fact, remarkable progresses have been made in pig immunology during the last years which has allow the use of these animals as clinical models of human diseases [28] including those related to the respiratory tract [29]. On the other hand, *L. gasseri* strains were assessed in this work considering that members of this species of the family *Lactobacillaceae* have been shown to possess immunomodulatory capacities and the ability to increase the resistance against viral infections. It was reported that orally administered *L. gasseri* SBT2055 is able to diminish the susceptibility to rotavirus infection by modulating the intestinal immune system [30]. Furthermore, the oral administration of *L. gasseri* SBT2055 was shown to protect mice against IFV [31] or RSV [32] infections in mice. Similar results were reported for orally administered *L. gasseri* TMC0356, originally isolated from human intestinal tract, that enhanced the resistance against IFV by the improvement of antiviral immunity in the respiratory tract [32]. To the best of our knowledge, no study has determined the capacity of nasally administered *L. gasseri* strains to modulate respiratory immunity or the interaction of alveolar macrophages with members of this species of lactobacilli. Thus, in the first set of experiments performed here we evaluated the ability of 36 *L. gasseri* strains to modulate the immune response of porcine alveolar macrophages (3D4/31 cells) to the stimulation with the TLR3 agonist poly(I:C). By determining the expression of *IFN-β* and *RNAseL* we selected *L. gasseri* TMT36, TMT39 and TMT40 as the strains with immunomodulatory potential.

Of note, viable immunobiotics are not strictly necessary to achieve immunomodulatory effects as some non-viable bacteria derived from them can exert similar protective effects [17,20,21,33]. Immunobiotics are by definition alive and required to have an efficacious number of viable bacteria at the time of administration to the host [34]. Thus, the maintenance of immunobiotics requires strict controls to maintain viability, which often results in large delays in their utilization due to the increased production and maintenance costs [22]. In contrast, the use of non-viable probiotics has practical advantages related to their utilization, although the potential influence of non-viable immunobiotics on antiviral immunity has had little attention. A postbiotic was recently defined as a “preparation of inanimate microorganisms and/or their components that confers a health benefit on the host” [35]. Here, we propose the term postimmunobiotic to designate non-viable bacteria derived from immunobiotics strains that retains their functionality. Postimmunobiotics represent an interesting alternative to modulate the respiratory immunity. In fact, our studies have reported that heat-killed *L. rhamnosus* CRL1505 has protective effects comparable to live bacteria [17,20,21,25]. In addition, it was reported that orally administered heat-killed *L. gasseri* TMC0356 is able to restore the expression levels of *IFN-γ* and *IL-2rb* induced by obesity in mice [33]. These previous works stimulated us to investigate the effect of heat-killed *L. gasseri* TMT36, TMT39 and TMT40 strains in the innate antiviral immune response in the respiratory tract. Our results demonstrated that postimmunobiotics derived from TMT36, TMT39 and TMT40 strains were capable of modulating the immune response triggered by TLR3 in porcine alveolar macrophages and to reduce lung inflammatory damage triggered by TLR3 activation in vivo.

In our hands, HK36, HK39 and HK40 modulated the expression of *IFN-β*, *IFN-λ1*, and the antiviral factors (*RNAseL* and *Mx2*) in porcine alveolar macrophages indicting their capacity to improve the innate antiviral immune response in these respiratory immune cell population. Alveolar macrophages are the first immune cells that encounter viruses that reach the deep lung and have a key role in the generation and regulation of immune responses against those pathogens [36]. Through the production of type I and type III IFNs, alveolar macrophages trigger the earliest host response that intends to eliminate virions and infected cells during respiratory virus infections [37,38,39,40]. IFNs released by alveolar macrophages act on immune and non-immune cells of the respiratory tract modulating the expression of antiviral factors such as RNAseL, Mx1, Mx2, and OAS molecules that contribute to viral clearance [36,41]. In addition, we observed that HK36, HK39 and HK40 treatments enhanced the expression of *IL-6* and *MCP-1* in porcine alveolar macrophages stimulated with poly(I:C). It was shown that type I IFNs production by alveolar macrophages up-regulate inflammatory cytokines and chemokines expression in the respiratory tract, which induce the recruitment of inflammatory monocytes/macrophages that contribute to the virus-infected cells clearance [41]. In line with these findings, our in vivo studies in mice showed that nasally administered HK36, HK39 and HK40 increased the levels of IFNs (IFN-β and IFN-λ) in the respiratory tract after the activation of TLR3. In addition, we observed that the nasal priming of mice with HK36, HK39 or HK40 significantly increased the levels of IFN-γ in the respiratory tract. It was shown that IFN-γ has a key protective role against respiratory viral infections [42,43]. An enhanced severe RSV-associated respiratory illness was found when alveolar macrophages were unable to produce appropriate amounts of IFN-γ [44,45]. Then, our data show that the efficient production of IFNs, antiviral factors, IL-1β and IL-6 by alveolar macrophages induced by HK36, HK39 and HK40 treatments could mediate increased protection against respiratory viral infections. Furthermore, our results indicate that the evaluation of lactobacilli and porcine alveolar macrophages interaction is a valuable in vitro tool to select promising immunobiotic and postimmunobiotic candidates. Our transcriptomic analysis successfully identified a group of genes (*IFN-β*, *IFN-λ1*, *RNAseL*, *Mx2,* and *IL-6*), which can be used as prospective biomarkers for the screening of new antiviral immunobiotics and postimmunobiotics in 3D4/31 cells. Our studies should be complemented with experiments with viral pathogens challenges to show that postimmunobiotics are effectively able to modulate the response of porcine alveolar macrophages to respiratory viruses such as RSV or IFV as well as to protect against infections in vivo. These are studies that we intend to perform in the immediate future.

Type I (IFN-α/β) and type III (IFN-λs) IFNs as well as IFN-γ play important roles in the resistance to respiratory viruses not only in human and mice but in addition in animals of economic importance like pigs [35,46]. In vitro studies with primary cultures of porcine alveolar macrophages demonstrated an enhanced capacity of these cells to inhibit porcine reproductive and respiratory syndrome virus (PRRSV) replication after their activation with IFN-γ [47]. The transcriptome evaluation of IFN-γ-treated porcine alveolar macrophages showed a significant up-regulation of inflammatory cytokines and chemokines (*GM-CSF2, GM-CSF3,* *CCL4*, *CCL20, CXCL2, CXCL9, CXCL20,* and *IL-12B*) as well as host restriction factors (*IFITM2*, *Mx1*, *Mx2*, *OASL* and *ISG15*) [48] that were associated to the increased resistance to PRRSV infection [49,50]. It was also demonstrated in monocyte-derived macrophages that PRRSV is able to inhibit the production of type I IFNs by antagonizing IRF3 activation [51]. In line with these results, it was described that porcine pulmonary alveolar macrophages produce negligible levels of IFN-α after the infection with PRRSV [52] while the treatment of cultured alveolar macrophages with IFN-α or IFN-β significantly reduce viral replication [53]. On the other hand, it was reported that IFN-λs also have protective functions against PRRSV infection. Dapulian pigs are recognized for their strong immune system and their resistance to pathogens [54]. Interestingly, it was reported that the expression of the IFN-λs receptor 1 (IFNLR1) gene in the lung of Dapulian pigs is significantly higher than in Landrace pigs and that this difference makes the former more resistant to PRRSV infection [55]. Then, the higher expression of *IRF3*, *IFN-β* and *IFN-λ1* found in porcine alveolar macrophages treated with HK36, HK39 or HK40 allow us to speculate that these postimmunobiotics could have beneficial effects in the protection of the respiratory tract against viral infections in the porcine host. Of note, type III IFNs consist of IFN-λ1 and IFN-λ3 in pigs [46]. It was reported that IFN-λ3 is a key factor with antiviral activity in primary cultures of porcine alveolar macrophages challenged with PRRSV [56,57]. The treatment of macrophages with IFN-λ3 enhanced the expressions of genes of the IFITM, MX and OAS families [56] reducing cytopathic effect and PRRSV replication [57]. Considering that HK36, HK39 or HK40 did not differentially modulate IFN-λ3 in 3D4/31 cells, deeper in vitro studies complemented by in vivo studies in pigs are necessary to evaluate the ability of these postimmunobiotics to protect against viral infections in these important agricultural animals.

Alveolar macrophages are also important for the regulation of the inflammatory damage during the course of respiratory viral infections. Comparative transcriptomic studies revealed that inflammatory cytokines are significantly enhanced in the lung of pigs infected with the virulent PRRSV XJ isolate compared to avirulent JS strain [58], and that the excessive pro-inflammatory cytokines production promote lung damage [59,60]. Similarly, it was shown that the impairment of the function of alveolar macrophages was associated with an exacerbated RSV-mediated respiratory disease [45]. In fact, alveolar macrophages depletion before the infection with RSV enhanced the recruitment of inflammatory cells (CD11b^hi^Gr1^hi^ neutrophils and CD11c^hi^MHC-II^hi^CD11b^+^ dendritic cells) to the lungs that contribute to a hyperresponsiveness in infected mice [43]. It was also shown that the absence of alveolar macrophages in *Csf2*^-/-^ mice resulted in severe morbidity to IFV infection [61]. In line with these findings, we demonstrated previously that the depletion of alveolar macrophages completely abolished the ability of the nasal priming with the postimmunobiotc NV1505 to protect against the lung inflammatory-mediated damage induced by TLR3 activation [20,21]. Furthermore, we showed that alveolar macrophages from mice nasally primed with NV1505 had an enhanced ability to produce IL-27 and IL-6 in response to the TLR3 activation. It was described that IL-27 helps in the control of the RSV infection severity by suppressing Th17- and Th2-mediated inflammations [62]. In addition, it was reported that IL-27 together with IL-6 are required for the stimulation of IL-10-producing Tregs [63]. Then, the data presented here indicate that similar to NV1505, the HK36, HK39 and HK40 treatments are able to enhance the ability of alveolar macrophages to secrete IL-27 and IL-6 limiting inflammation and protecting lung function during the activation of TLR3 by increasing the activation of IL-10-producing Treg cells.

We demonstrated previously that the ability of immunobiotics strains to modulate the response of intestinal epithelial cells to the activation of TLR3 was mediated by their ability to induce changes in the expressions of negative regulators of the TLR signaling pathways [24,25]. The results of this work indicate that the modulation of TLR negative regulators would be also important in the ability of postimmunobiotics to modulate the innate antiviral response of alveolar macrophages. Here, we observed that the treatment of porcine alveolar macrophages with poly(I:C) significantly enhanced the expression levels of *Tollip, A20, SIGIRR* and *IRAK-M* which is in line with previous reports showing the rapid up-regulation of *Tollip*, *A20* and *IRAK-M* in human alveolar macrophages after poly(I:C) stimulation [64]. This transcriptomic response of macrophages would be related to their role in protecting against lung damage mediated by the inflammatory response during viral infectious processes. In fact, it was reported that macrophages deficient in *A20* expression are hyperresponsive to dsRNA stimulation and IFV infection since they respond with higher levels of NF-κB and IRF3 activation, and the subsequent enhanced production proinflammatory cytokines and chemokines. *In vivo, A20* myeloid cell specific deficiency was associated with an increased number of neutrophils in the lungs of IFV infected mice [65]. We also demonstrated that HK36, HK39 and HK40 increased the expression of *A20* in porcine alveolar macrophages stimulated with poly(I:C). The function of negative regulators is of fundamental importance to control the magnitude and duration of TLR signalling [66]. A20, encoded by the *TNFAIP3* gene, has been documented to inhibit TLR signaling in macrophages through its action on TRAF6 impeding its binding to TAB2 and TAB3, the recruitment of TAK1 and thereby, reducing NF-κB activation [66]. Furthermore, A20 expressed in alveolar macrophages has been shown to be key for the control of lung injury induced by LPS [50]. Some lactobacilli were reported to modulate macrophage TLR4-mediated inflammatory responses through the modulation of the expression of A20. It was shown that the treatment of differentiated THP-1 cells with *Lacticaseibacillus paracasei* reduced the LPS-induced production of the inflammatory cytokines TNF-α and IL-1β, and that this effect was related mainly to a decrease of NF-κB activation by the up-regulation of *A20* [67]. Similarly, *Lactobacillus helveticus* SBT2171 LH2171 diminished LPS-induced secretion of IL-6 and IL-1β by increasing *A20* [68]. Of note, HK36, HK39 and HK40 decreased the expression of *Tollip* in porcine alveolar macrophages stimulated with poly(I:C). Considering that Tollip interacts with IRAK1 and inhibits the subsequent TRAF6 activation [69], it could be speculated that this mechanism could be involved in the enhancement of the production of inflammatory cytokines by macrophages induced by the postimmunobiotics treatments. The role of negative regulators of the TLR signaling pathway in the ability of immunobiotics to modulate TLR3-mediated inflammation in macrophages has not been well studied. To the best of our knowledge, our study is the first one to investigate the expression of *A20* and *Tollip* in the response of porcine alveolar macrophages to the stimulation of poly(I:C) and the first to suggest their role in the ability of immunobiotics to improve the anti-inflammatory and protective effects of respiratory macrophages.

An important question that arises from our results is which molecule(s) present in the postimmunobiotics HK36, HK39 and HK40 can regulate the TLR3 signalling pathway in alveolar macrophages. One possibility is that RNA molecules derived from lactobacilli act directly on TLR3, modifying its subsequent response to poly(I:C) stimulation. In this regard, it was shown that double-stranded RNA (dsRNA) derived from some lactic acid bacteria strains modulate IFNs and cytokine expression in antigen presenting cells [70]. Interestingly, it was reported that LAB derived dsRNA molecules were resistant to heat treatment indicating that they could be present in postimmuobiotics [70]. Another possibility is that postimmunobiotics modulate the TLR3 signalling pathway through other PRRs such as TLR2. Some works have demonstrated that cell wall components of lactobacilli such as peptidoglycan can stimulate TLR2 in macrophages inducing changes in their expression of immune factors [71,72,73]. Of note, the activation of TLR2 pathway by lactobacilli can induce a differential expression of regulators of PRRs such as A20 thereby indirectly influencing the TLR4 signalling pathway. It was shown that *Lactobacillus helveticus* SBT2171 induces A20 expression via TLR2 and inhibits the LPS-induced activation of NF-kB and MAPK in peritoneal macrophages [68]. Our own studies in porcine mononuclear phagocytes from Peyer’s patches demonstrated that *Lactobacillus jensenii* TL2937 activate TLR2 and differentially regulated the subsequent TLR4 activation by modulating TLRs negative regulators [72,73]. Therefore, the possibility that HK36, HK39 and HK40 act through TLR2 inducing regulators that then influence the TLR3 pathway in alveolar macrophages is a hypothesis that should be studied.

## 5. Conclusions

In conclusion, we have provided evidence that porcine alveolar macrophages (3D4/31 cells) are a useful in vitro tool for the screening of new antiviral immunobiotics and postimmunobiotics by assessing their ability to modulate the expression *IFN-β*, *IFN-λ1*, *RNAseL*, *Mx2,* and *IL-6*, which can be used as prospective biomarkers. We also demonstrated that the postimmunobiotics derived from the *L. gasseri* TMT36, TMT39 and TMT40 strains modulate the innate antiviral immune response of alveolar macrophages and the TLR3-mediated respiratory immunity in vivo. Although our findings should be deepened and expanded, the results of the present work provide a scientific rationale for the use of nasally administered HK36, HK39 or HK40 to beneficially modulate TLR3-triggerd respiratory innate immune response.

## Data Availability

Data is contained within the article.

## Supplementary Materials

The following supporting information can be downloaded at: https://www.mdpi.com/article/10.3390/cells11192986/s1, Table S1: Primer sequences for RT-qPCR in porcine alveolar macrophages; Table S2: Lactic acid bacteria strains used in this work.
