# Supplementary material for: Modulation of Alveolar Macrophages by Postimmunobiotics: Impact on TLR3-Mediated Antiviral Respiratory Immunity
Source: Cells. 2022 Sep 25;11(19):2986. doi: 10.3390/cells11192986 (PMC9562200; doi:10.3390/cells11192986)
Supplement: Supplementary file 1 [file cells-11-02986-s001.zip › cells-1869885-Table S1 Primer sequence.pdf]

Table S1. Primer sequences for RT-qPCR in porcine alveolar macrophages

Target	Forward primer	Reverse primer
IFN-α	CAGGGCAGAAGTCATGAGATCC	ATGAACCAGGTGTCTGTCACTC
IFN-β	AGTTGCCTGGGACTCCTCAA	CCTCAGGGACCTCAAAGTTCAT
IFN-λ1	CCTTAGAGGCTGAGCTAGACTTGAC	AGCCTGAAGTTCGACGTGGATG
IFN-λ3	GTTCAAGTCTCTGTCCCCACAAG	CTCCAAGAGGGACTCTTCAAAGG
RNase L	AGTGGACAACTAAGATTGACAG	GTAGACATACATGACCAGATCG
Mx1	GAGGTGGACCCCGAAGGA	CACCAGATCCGGCTTCGT
Mx2	AATCATCACCAGGTGTCCGC	CTTTGCGTATTTCCCGCTCC
MDA-5	GCTACGTGAACCCCGATCTC	AAGCTTGTCCACCACTGTAGG
RIG-I	TATCCGAGCAGCAGGCTTTG	CTCGTTGCTGGGATCTATGGAA
PKR	CCCTGCACTTCTAGCCATCT	CGACCACTGGCCATTTCTTTC
IL-6	TGGATAAGCTGCAGTCACAG	ATTATCCGAATGGCCCTCAG
MCP-1	ACAGAAGAGTCACCAGCAGCAA	GCCCGCGATGGTCTTG
Tollip	TACCGTGGGCCGTCTCA	CCGTAGTTCTTCGCCAACTTG
A20	CCTCCCTGGAAAGCCAGAA	GTGCCACAAGCTTCCTCACTT
MKP-1	TCCGAATTCCACTGGGTTCC	ATGTTGGTCCCCAATGTGCT
Bcl-3	CGACGCGGTGGAC. ATTAAG	ACCATGCTAAGGCTGTTGTTTTC
SIGIRR	ATGTGAAGTGTCGGCTCAATGT	TTCATCTCCACCTCCCCATACT
IRAK-M	TGGAGCAGCCTTGAATCCTT	TGGATAACACGTTTGGGAATCTT
β-actin	CATCACCATCGGCAACGA	GCGTAGAGGTCCTTCCTGATGT
