# Supplementary material for: Modulation of Alveolar Macrophages by Postimmunobiotics: Impact on TLR3-Mediated Antiviral Respiratory Immunity
Source: Cells. 2022 Sep 25;11(19):2986. doi: 10.3390/cells11192986 (PMC9562200; doi:10.3390/cells11192986)
Supplement: Supplementary file 1 [file cells-11-02986-s001.zip › cells-1869885-Table S2 strains.pdf]

Table S2. Lactic acid bacteria strains used in this work.

	**Organism name**	**Strain**	**Origin**
1	*Lactobacillus gasseri*	TMT1	Human feces
2	*Lactobacillus gasseri*	TMT2	Human feces
3	*Lactobacillus gasseri*	TMT3	Human feces
4	*Lactobacillus gasseri*	TMT4	Human feces
5	*Lactobacillus gasseri*	TMT5	Human feces
6	*Lactobacillus gasseri*	TMT6	Human feces
7	*Lactobacillus gasseri*	TMT7	Human feces
8	*Lactobacillus gasseri*	TMT8	Human feces
9	*Lactobacillus gasseri*	TMT9	Human feces
10	*Lactobacillus gasseri*	TMT11	Human feces
11	*Lactobacillus gasseri*	TMT12	Human feces
12	*Lactobacillus gasseri*	TMT13	Human feces
13	*Lactobacillus gasseri*	TMT14	Human feces
14	*Lactobacillus gasseri*	TMT15	Human feces
15	*Lactobacillus gasseri*	TMT16	Human feces
16	*Lactobacillus gasseri*	TMT17	Human feces
17	*Lactobacillus gasseri*	TMT18	Human feces
18	*Lactobacillus gasseri*	TMT19	Human feces
19	*Lactobacillus gasseri*	TMT20	Human feces
20	*Lactobacillus gasseri*	TMT21	Human feces
21	*Lactobacillus gasseri*	TMT22	Human feces
22	*Lactobacillus gasseri*	TMT23	Human feces
23	*Lactobacillus gasseri*	TMT24	Human feces
24	*Lactobacillus gasseri*	TMT25	Human feces
25	*Lactobacillus gasseri*	TMT26	Human feces
26	*Lactobacillus gasseri*	TMT27	Human feces
27	*Lactobacillus gasseri*	TMT28	Human feces
28	*Lactobacillus gasseri*	TMT29	Human feces
29	*Lactobacillus gasseri*	TMT31	Human feces
30	*Lactobacillus gasseri*	TMT32	Human feces
31	*Lactobacillus gasseri*	TMT34	Human feces
32	*Lactobacillus gasseri*	TMT35	Human feces
33	*Lactobacillus gasseri*	TMT36	Human feces
34	*Lactobacillus gasseri*	TMT38	Human feces
35	*Lactobacillus gasseri*	TMT39	Human feces
36	*Lactobacillus gasseri*	TMT40	Human feces
